# Predicting Patterns of Long-Term CD4 Reconstitution in HIV-Infected Children Starting Antiretroviral Therapy in Sub-Saharan Africa: A Cohort-Based Modelling Study

**DOI:** 10.1371/journal.pmed.1001542

**Published:** 2013-10-29

**Authors:** Marie-Quitterie Picat, Joanna Lewis, Victor Musiime, Andrew Prendergast, Kusum Nathoo, Addy Kekitiinwa, Patricia Nahirya Ntege, Diana M. Gibb, Rodolphe Thiebaut, A. Sarah Walker, Nigel Klein, Robin Callard, the ARROW Trial Team

**Affiliations:** 1 Institute of Child Health, University College London, London, United Kingdom; 2 Institut de Santé Publique, d'Épidémiologie et de Développement, Centre Inserm U897–Epidemiologie-Biostatistique, University of Bordeaux, Bordeaux, France; 3 Department of Medical Information, Bordeaux University Hospital, Bordeaux, France; 4 Centre for Mathematics and Physics in the Life Sciences and Experimental Biology, University College London, London, United Kingdom; 5 Joint Clinical Research Centre, Kampala, Uganda; 6 MRC Clinical Trials Unit, Medical Research Council, London, United Kingdom; 7 Centre for Paediatrics, Blizard Institute, Queen Mary University of London, London, United Kingdom; 8 University of Zimbabwe Medical School, University of Zimbabwe, Harare, Zimbabwe; 9 Paediatric Infectious Diseases Centre, Mulago, Uganda; 10 MRC/UVRI Uganda Research Unit on AIDS, Uganda Virus Research Institute, Entebbe, Uganda; San Francisco General Hospital, United States of America

## Abstract

**Background:**

Long-term immune reconstitution on antiretroviral therapy (ART) has important implications for HIV-infected children, who increasingly survive into adulthood. Children's response to ART differs from adults', and better descriptive and predictive models of reconstitution are needed to guide policy and direct research. We present statistical models characterising, qualitatively and quantitatively, patterns of long-term CD4 recovery.

**Methods and Findings:**

CD4 counts every 12 wk over a median (interquartile range) of 4.0 (3.7, 4.4) y in 1,206 HIV-infected children, aged 0.4–17.6 y, starting ART in the Antiretroviral Research for Watoto trial (ISRCTN 24791884) were analysed in an exploratory analysis supplementary to the trial's pre-specified outcomes. Most (*n* = 914; 76%) children's CD4 counts rose quickly on ART to a constant age-corrected level. Using nonlinear mixed-effects models, higher long-term CD4 counts were predicted for children starting ART younger, and with higher CD4 counts (*p*<0.001). These results suggest that current World Health Organization–recommended CD4 thresholds for starting ART in children ≥5 y will result in lower CD4 counts in older children when they become adults, such that vertically infected children who remain ART-naïve beyond 10 y of age are unlikely ever to normalise CD4 count, regardless of CD4 count at ART initiation. CD4 profiles with four qualitatively distinct reconstitution patterns were seen in the remaining 292 (24%) children. Study limitations included incomplete viral load data, and that the uncertainty in allocating children to distinct reconstitution groups was not modelled.

**Conclusions:**

Although younger ART-naïve children are at high risk of disease progression, they have good potential for achieving high CD4 counts on ART in later life provided ART is initiated following current World Health Organization (WHO), Paediatric European Network for Treatment of AIDS, or US Centers for Disease Control and Prevention guidelines. In contrast, to maximise CD4 reconstitution in treatment-naïve children >10 y, ART should ideally be considered even if there is a low risk of immediate disease progression. Further exploration of the immunological mechanisms for these CD4 recovery profiles should help guide management of paediatric HIV infection and optimise children's immunological development.

*Please see later in the article for the Editors' Summary*

## Abstract 2

Using data from the ARROW trial, Joanna Lewis and colleagues investigate the CD4 cell count recovery profiles of children infected with HIV starting antiretroviral therapy in Sub-Saharan Africa.

*Please see later in the article for the Editors' Summary*

## Editors' Summary

**Background:**

Worldwide, about 3.3 million children under 15 years old are infected with HIV, the virus that causes AIDS. More than 90% of these children live in sub-Saharan Africa, where nearly 600 children become infected with HIV every day, usually acquiring the virus from their mother during pregnancy, birth, or breastfeeding. HIV gradually reduces the numbers of CD4 lymphocytes in the immune system, leaving infected individuals susceptible to other infections. HIV infection can be kept in check but not cured with antiretroviral therapy (ART)—cocktails of drugs that have to be taken every day throughout life. ART reduces the amount of virus in the blood (viral load), which allows the immune system to recover (long-term immune reconstitution). Unfortunately, ART is very expensive, but concerted international efforts over the past decade mean that about a third of children who need ART are now receiving it, including half a million children in sub-Saharan Africa.

**Why Was This Study Done?:**

World Health Organization (WHO) guidelines recommend initiation of ART at age-related CD4 cell count thresholds based on the risk of short-term disease progression. The guidelines recommend that all HIV-positive children under two years old begin ART as soon they receive a diagnosis of HIV infection. For children aged 2–5 years, ART initiation is recommended once the CD4 count drops below 750 cells/µl blood, whereas for older children the threshold for ART initiation is 350 CD4 cells/µl. Because of improved ART coverage, many more HIV-infected children now survive into adulthood than in the past. It is therefore important to know how the timing of ART initiation in childhood affects long-term immune reconstitution. Unfortunately, although several studies have examined the effect of ART on immune reconstitution in adults, the results of these studies cannot be extrapolated to children because of age-related differences in immune reconstitution. In this cohort-based modelling study, the researchers investigate long-term CD4 recovery in a cohort (group) of HIV-infected children initiating ART in Uganda and Zimbabwe, and present statistical models that predict patterns of long-term CD4 status based on age and CD4 count at ART initiation.

**What Did the Researchers Do and Find?:**

To investigate long-term CD4 reconstitution in children, the researchers used CD4 counts collected during the ARROW trial, a study designed to investigate monitoring strategies during first-line ART in 1,206 HIV-positive children. In three-quarters of the children, CD4 reconstitution following ART initiation was asymptotic—CD4 counts increased rapidly immediately after ART initiation, then slowed before eventually reaching a constant level of about 80% of the CD4 count expected in an uninfected child of the same age. Using a nonlinear mixed-effects statistical model that fitted this pattern of immune reconstitution, the researchers predicted CD4 trajectories for children starting ART at different ages and with different CD4 counts. Higher long-term counts were predicted for children starting ART earlier and with higher CD4 counts. Thus, to achieve a CD4 count greater than 700 cells/µl at age 20 years, CD4 counts of at least 96 cells/µl, 130 cells/µl, and 557 cells/µl are needed for children aged two, five, and 12 years, respectively, when they initiate ART. Qualitatively distinct reconstitution patterns were seen in the remaining children in the study.

**What Do These Findings Mean?:**

These findings suggest that young HIV-positive, ART-naïve children can achieve high CD4 counts in later life, provided ART is initiated as recommended in the current WHO guidelines. However, the recommended CD4 count thresholds for ART initiation are unlikely to maximize immune reconstitution in treatment-naïve children over ten years old. Rather, these findings suggest that ART initiation should be considered in these older children when their CD4 count is still high—even though they have a low risk of immediate disease progression—in order to achieve higher long-term CD4 levels. The omission of viral load measurements in the researchers' model may limit the accuracy of these findings. Moreover, although the predictions made by the model apply to children who will go on to experience asymptotic recovery, they are less relevant to those with different recovery profiles, who cannot currently be accurately identified. Further exploration of the immunological mechanisms underlying the CD4 recovery profiles described here should improve our understanding of the factors that determine the response of HIV-positive children to ART and provide information to guide the management of HIV infections in children.

**Additional Information:**

Please access these websites via the online version of this summary at http://dx.doi.org/10.1371/journal.pmed.1001542.

- Information is available from the US National Institute of Allergy and Infectious Diseases on HIV infection and AIDS
- NAM/aidsmap provides basic information about HIV/AIDS and summaries of recent research findings on HIV care and treatment
- Information is available from Avert, an international AIDS charity, on many aspects of HIV/AIDS, including information on HIV and AIDS in Africa and on HIV infection in children (in English and Spanish)
- The UNAIDS World AIDS Day Report 2012 provides up-to-date information about the AIDS epidemic and efforts to halt it; the UNAIDS's 2013 Progress Report on the Global Plan provides information on progress towards eliminating new HIV infections among children by 2015
- The World Health Organization provides information about universal access to AIDS treatment (in several languages); its 2010 guidelines for ART in infants and children can be downloaded
- Information about the ARROW trial is available
- Personal stories about living with HIV/AIDS, including stories from young people infected with HIV, are available through Avert, through Nam/aidsmap, and through the charity website Healthtalkonline

## Introduction

By December 2011, an estimated 3.3 million children under 15 y of age were living with HIV worldwide, over 90% of whom lived in sub-Saharan Africa [1]. While the 28% coverage of antiretroviral therapy (ART) for children in need lags behind the 58% coverage in adults, the number of HIV-infected children receiving ART has nevertheless increased to around 563,000, of whom 88% are in sub-Saharan Africa. Most receive ART under a public health approach with little, if any, laboratory monitoring [2],[3]. Current guidelines recommend initiation of ART at age-related CD4 thresholds that are based on the risk of short-term disease progression. However, with increasing likelihood of survival into adulthood, a major challenge is to better understand how timing of ART initiation in childhood influences long-term immune reconstitution and immunological health [3]. To date, studies describing ART outcomes in resource-limited settings have usually reported only short-term (<1–2 y) disease progression and CD4 changes as outcomes [2],[4]. Studies of longer-term immune reconstitution after ART initiation are scarce, and have typically included fewer than 200 children [5]–[8].

Although some large studies have assessed the long-term immunological effects of ART in adults in resource-limited settings [9],[10], immune reconstitution differs significantly in children [11]–[14], most likely as a result of their more active thymopoiesis [15]–[17]. Successful approaches for studying long-term immune reconstitution in adults [18], and the conclusions drawn, may therefore not extend to children, and particularly not across all age groups in childhood. In addition, factors such as greater burden of co-infections and higher rates of malnutrition may result in differences in CD4 response between children in well-resourced and resource-limited settings.

We previously developed a statistical model for CD4 reconstitution in 127 European/South American HIV-infected children starting ART [19]. It assumes that the rate of increase in CD4 count (standardised for age) is fastest immediately following treatment initiation, falling exponentially with treatment duration so that CD4 count tends to a constant value in the long term, ideally approaching the count in a healthy child of the same age. By incorporating factors including age at treatment initiation in our model, we found that current guidelines for ART initiation, which are designed to reduce short-term risk of disease progression, may not always be optimal in terms of long-term immune reconstitution.

With the aim of understanding the kinetics of long-term CD4 reconstitution, we have investigated a large cohort of HIV-infected, ART-naïve children initiating ART in Uganda and Zimbabwe and followed up for a median (interquartile range [IQR]) 4.0 (3.7, 4.4) y. We identified qualitatively different CD4 recovery profiles using linear and nonlinear regression. We also present predictive models of long-term CD4 status based on age and CD4 count at ART initiation, constructed using nonlinear mixed-effects models. By taking into consideration long-term immunological health, this approach should better inform decisions for ART initiation, which to date have been based mainly on risks of short-term disease progression in untreated children before the era of ART.

## Methods

### Participants and Follow-Up

We used data from the Antiretroviral Research for Watoto (ARROW) trial, an open-label, randomised 5-y paediatric trial comparing two monitoring strategies during first-line ART (http://www.arrowtrial.org/; ISRCTN 24791884 [20]). 1,206 HIV-infected, ART-naïve children aged 3 mo to 18 y and meeting WHO criteria for ART were enrolled at three centres in Uganda (Joint Clinical Research Centre, Paediatric Infectious Diseases Centre, and Medical Research Council/Uganda Virus Research Institute Uganda Research Unit on AIDS) and one in Zimbabwe (University of Zimbabwe, Harare). The trial was approved by research ethics committees in Uganda, Zimbabwe, and the United Kingdom. Written informed consent was obtained from children's parents/guardians, and written assent from the children as appropriate.

The ARROW trial's primary aim was to compare two monitoring strategies in children starting ART. Accordingly, children were randomised to either the “clinically driven monitoring” (CDM) or “laboratory and clinical monitoring” (LCM) group. In both groups, children had a routine full blood count, lymphocyte subsets (CD4, CD8), and liver and renal function tests (bilirubin, urea, creatinine, aspartate transaminase, alanine transaminase) performed every 12 wk. Demographic (age, sex) and clinical (WHO staging, height, weight) data were also collected. An additional week 4 lymphocyte subset measurement was available in children participating in an immunology substudy. In the CDM group, test results were returned to the clinician only if he or she requested them for clinical reasons, or for a grade 4 adverse event as defined in the trial protocol [21]. No total lymphocyte or CD4 counts were ever returned for CDM participants. Independently of monitoring randomisation, all children were also randomised to one of three first-line ART strategies. A control group (Arm A) received two nucleoside reverse transcriptase inhibitor (NRTI) drugs (lamivudine and abacavir) with one non-nucleoside reverse transcriptase inhibitor (NNRTI)—nevirapine in children aged <3 y and either nevirapine or efavirenz for older children based on clinician choice—according to WHO guidelines. The remaining children received these three drugs and also a third NRTI, zidovudine. After 36 wk, children who had started four-drug ART dropped either zidovudine (Arm B) or their NNRTI (nevirapine or efavirenz; Arm C). Criteria for failure of first-line ART and switch to second-line ART were clinical in both monitoring groups, or immunological in the LCM group only, and followed WHO guidelines [3]. Participants were followed under these monitoring/ART strategies until the end of the overarching ARROW trial on 16 March 2012.

No HIV viral loads were assayed in real time, following national guidelines, although samples were stored, and retrospective testing was carried out at a subset of time points and in a subset of children to address specific focussed questions [20]. The sampling design involved running assays for samples taken at 0, 4, 24, 36, 48, and 144 wk after ART initiation in all children aged <5 y at enrolment, plus in older children enrolled after 1 June 2008 and participating in the immunology substudy (assays 70% complete). Samples were also assayed at the time of a secondary randomisation to once versus twice daily lamivudine+abacavir, and 48 and 96 wk subsequently (assays 95% complete), and at 36, 48, and 84 wk in 41 children enrolled in a pharmacokinetic substudy of once versus twice daily lamivudine+abacavir (assays 100% complete). One centre also systematically performed viral load assays in all children as they exited the trial (i.e., did not select according to possible failure). In children for whom some post-enrolment viral loads were available, we characterised virological response to ART by calculating the mean log_10_ viral load (in RNA copies/millilitre) (“average log-viral load”) and the proportion of a child's viral load measurements that were above 80 RNA copies/ml (“proportion of viral loads detectable”).

The primary and secondary randomised comparisons have been reported separately [20]. This analysis is exploratory, was not specified in the protocol, and was conducted with the aim of understanding and modelling qualitatively different patterns of immune reconstitution.

### Age-Adjusted CD4 Count

CD4 T cell counts were adjusted for age by dividing by the CD4 count that would be expected in HIV-uninfected children of the same age [22],[23]. Thus, a ratio (“CD4-for-age”) of 1 indicates the expected CD4 count for age, and ratios <1 indicate a lower than expected CD4 count for age. The CD4 *z*-score (normalised centile of the observed CD4 count within a reference population of uninfected, HIV-exposed children of the same age) [24] was not used here because *z*-scores do not allow the youngest children to have extremely low scores (e.g., 1 CD4 cell/µl has *z*-score −8.1 in a 1-y-old, but −62 in a 10-y-old), and the *z*-score is also very sensitive to small errors in CD4 count at low values (Figure S1). Although CD4 percentage is more stable with age and has been widely used [25],[26], CD4 count has been shown to have greater prognostic value [27] and so was the preferred variable in this analysis.

### Statistical Analyses

**Figure 1:**
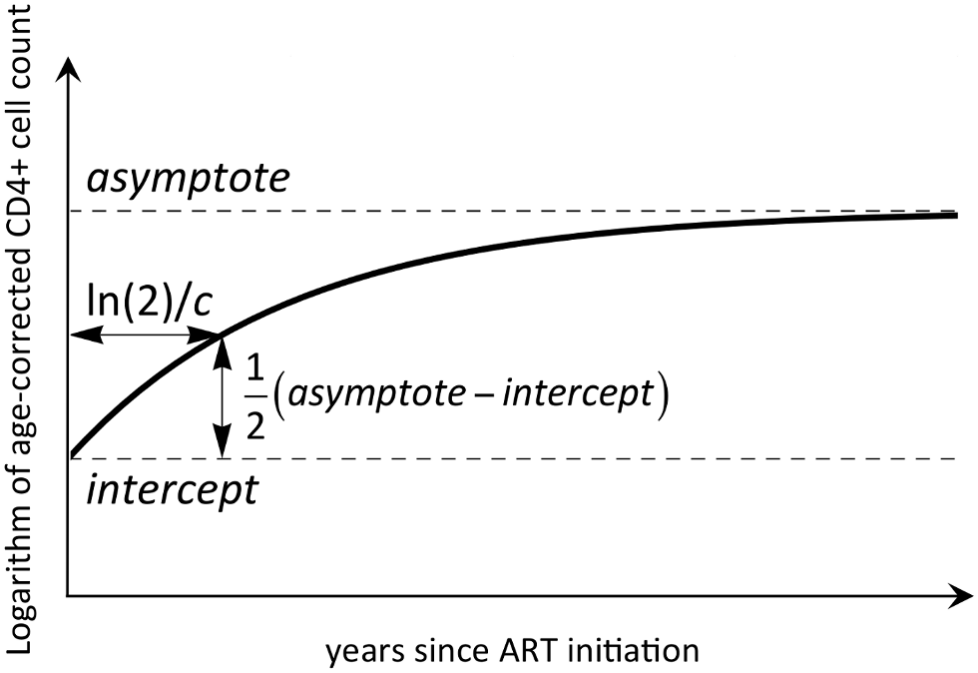
An illustrative representation of the mathematical model used to describe CD4 reconstitution.

Our mathematical model, which assumes nonlinear CD4-for-age recovery over time, tending towards a final stable level (asymptote) in the long term, has been previously described [19]. It is summarised as follows for CD4-for-age in child *i* at time *t_ij_*:
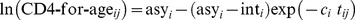
(1)The child-specific intercept (int*_i_*) is the ln(CD4-for-age) value at treatment initiation. The asymptote (asy*_i_*) is the long-term ln(CD4-for-age) value. The proportional recovery rate *c_i_* characterises the time for recovery, with ln(2)/*c* being the time for half the total recovery from int to asy to occur (illustrated in Figure 1). Transformation of CD4-for-age onto the log scale improves normality.

#### Identifying children with asymptotic CD4 reconstitution profiles

In preliminary analysis we found that the model did not converge onto the entire dataset. To identify children whose CD4-for-age trajectories followed Equation 1 (“asymptotic reconstitution”), each child's CD4 counts were modelled individually, using least squares to fit each child's data to Equation 1. Children were divided into two groups: one in which the least-squares fit had converged, and the other in which it had not. The first group thus consisted of children with asymptotic reconstitution, while the other comprised children with qualitatively different CD4 reconstitution profiles.

#### Modelling asymptotic reconstitution profiles

In the group with asymptotic CD4 reconstitution, recovery was analysed using nonlinear mixed-effects models for ln(CD4-for-age), based on Equation 1. The mixed-effects model assumes that in each child the values of the three parameters int, asy, and *c* are the sum of a fixed effect—which describes the average response in the whole population of children with asymptotic reconstitution—and a normally distributed random effect—which accounts for and describes inter-individual variability. A normally distributed error term ε*_ij_* is added to Equation 1 to represent noise, measurement error, and model mis-specification.

We investigated the predictive value of age, sex, WHO disease stage, height- and weight-for-age *z*-scores at ART initiation, and randomisation to monitoring and first-line ART strategies on proportional recovery rate (*c*) and CD4-for-age at ART initiation (int) and in the long term (asy), using a backwards-selection procedure based on an exit *p* = 0.01 to favour parsimony and avoid including small significant but clinically unimportant effects given the size of the dataset. Height- and weight-for-age *z*-scores were calculated using UK norms, which, unlike WHO references, cover the whole childhood age range [28].

#### Characterising non-asymptotic reconstitution

In children without asymptotic CD4 reconstitution, we used linear models to fit CD4-for-age trajectories and identify those with increasing or decreasing CD4-for-age, those in whom CD4-for-age did not change significantly, and those with insufficient data to characterise a recovery profile.

Analyses were performed in R [29], using the nlme package [30] for nonlinear modelling. Graphs interpreting the fitted parameter values in terms of predicted CD4 trajectories were produced using Mathematica [31].

## Results

1,206 ART-naïve children (51% girls) were enrolled in the ARROW trial between 15 March 2007 and 18 November 2008 and followed for a median (IQR) 4.0 (3.7, 4.4) y. 806 (67%) were from Uganda; 400 (33%) were from Zimbabwe. There were 600 children in the LCM group versus 606 children in the CDM group in the monitoring randomisation, and 397 children in Arm A (198 LCM, 199 CDM) versus 404 in Arm B (201 LCM, 203 CDM) versus 405 in Arm C (201 LCM, 204 CDM) in the ART strategy randomisation.

Median (IQR) age at ART initiation was 6.0 (2.4, 9.3) (range: 0.4–17.6) y, and the median (IQR) CD4 count and CD4-for-age were 360 (164, 694) cells/µl and 0.28 (0.14, 0.43), respectively. At ART initiation, 683 (57%) and 169 (14%) children were at WHO disease stage 3 and 4, respectively. Children were moderately wasted (median [IQR] weight-for-age *z*-score −2.2 [−3.3, −1.3]) and stunted (median [IQR] height-for-age *z*-score −2.4 [−3.4, −1.5]).

At least one post-enrolment viral load measurement was available in 950 children (79%), who had a median (IQR) age of 4.4 (2.0, 7.7) y, a median CD4 count of 439 (197, 780) cells/µl, and a median CD4-for-age of 0.29 (0.16, 0.46) at ART initiation. In these 951 children, the median (IQR) number of measurements per child was 4 (3, 7), and samples were collected after a median (IQR) 84 (36, 144) wk on ART.

### Contrasting Long-Term Profiles of CD4 Reconstitution Following ART Initiation

**Figure 2:**
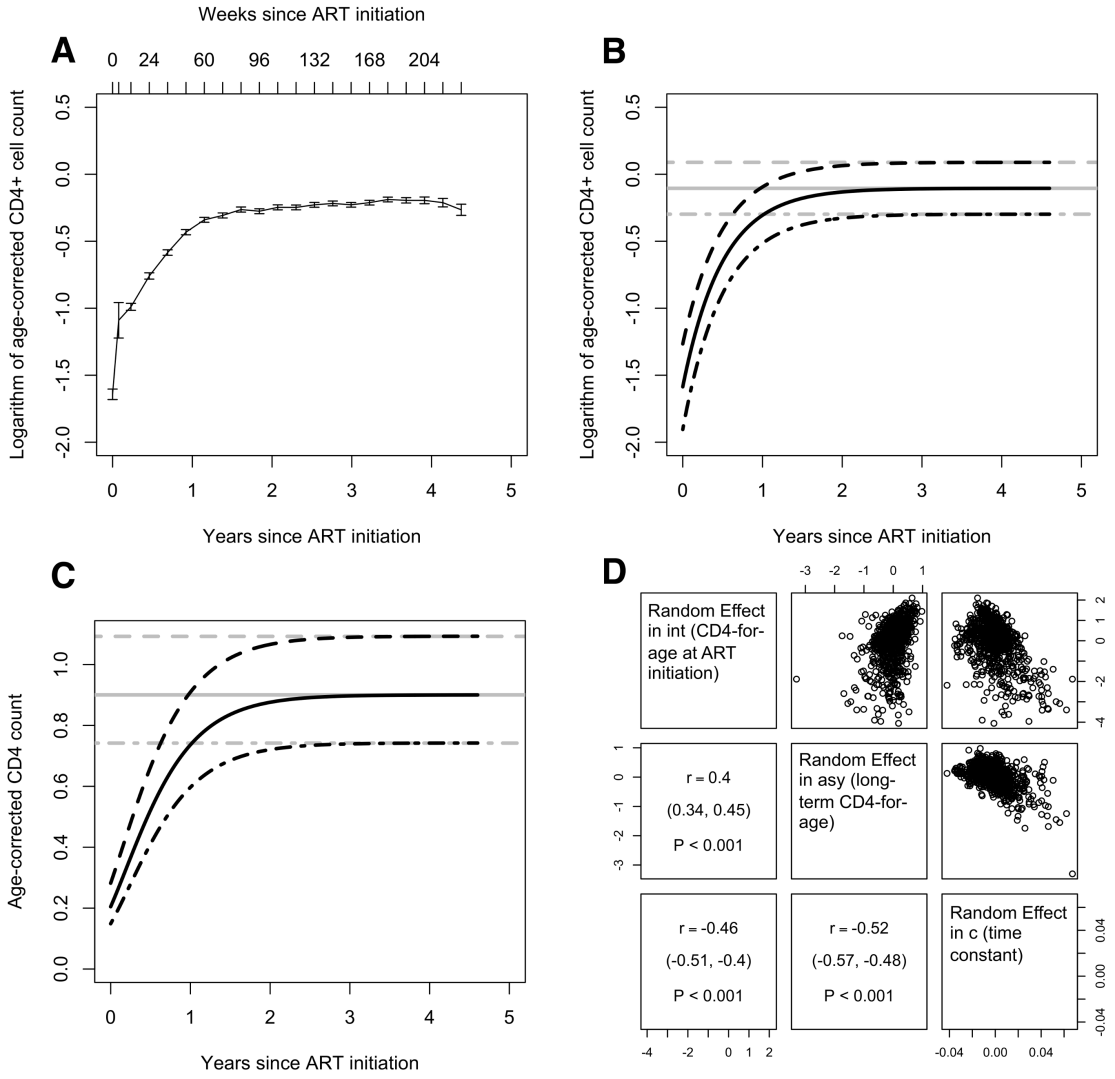
Modelling CD4-for-age in children showing asymptotic reconstitution. (A) Mean CD4-for-age over time since ART initiation for 914 children with asymptotic CD4 reconstitution. Errors bars give standard error of the mean. Points are shown for times where >20% of children in this group remained in the trial. Only children participating in a substudy had measurements available at week 4, accounting for the wider error bar. The numbers of children with measurements available at 0, 4, 12, 24, 36, 48, 60, 72, 84, 96, 108, 120, 132, 144, 156, 168, 180, 192, 204, 216, and 228 wk were 914, 153, 897, 893, 900, 893, 894, 898, 989, 900, 902, 898, 899, 897, 896, 892, 861, 790, 516, 354, and 232, respectively. (B) Population-average (fixed-effects) ln(CD4-for-age) trajectories predicted by our nonlinear mixed-effects model (Table 2) for “average” HIV-infected children (female, weight-for-age *z*-score at ART initiation −2.2) aged 2 (dashed line), 6 (solid line), and 10 (dot-dashed line) y. (C) Predicted CD4-for-age trajectories as shown in (B), but plotted on the linear scale (i.e., CD4/expected CD4). (D) Correlations between child-level random effects (difference from population average) in the three model parameters. Each panel refers to correlations between the estimated random effects (differences between child-specific parameters and the population average) in two of the three parameters int (CD4-for-age at ART initiation), asy (long-term CD4-for-age), and *c* (proportional recovery rate). The pair of parameters being considered is identified by the row and column labels in the panels on the diagonal. The upper-right panels plot the values of the random effects against one another. The lower-left panels give Pearson correlations between the estimated random effects, with 95% confidence intervals and *p*-values.

Our model identified two distinct groups according to long-term CD4 reconstitution profiles on ART. The first group included 914 (76%) children with asymptotic CD4 reconstitution (average response in this group shown in Figure 2B). Among the remaining 292 (24%) children, CD4-for-age values followed qualitatively different trajectories. At ART initiation, children with asymptotic reconstitution were younger (*p*<0.001) and had lower CD4-for-age (*p*<0.001) and height-for-age *z*-score (*p*<0.001) (Table 1). In multivariable logistic models, experiencing asymptotic reconstitution was independently associated with being younger at ART initiation (odds ratio [OR] [95% CI] = 0.94 [0.91, 0.97] per year older, *p*<0.001) and having lower height-for-age (OR = 0.86 [0.78, 0.95] per unit higher height-for-age, *p* = 0.003). Children randomised to four-drug, two-class induction followed by two-NRTI+NNRTI maintenance (Arm B) were more likely to have asymptotic reconstitution than the standard-of-care arm (Arm A) (OR = 1.48 [1.05, 2.07] higher, *p* = 0.024), but there was no evidence of a difference between three-NRTI maintenance (Arm C) and Arm A (OR = 0.93 [0.68, 1.28] higher, *p* = 0.65). Notably, monitoring strategy (CDM versus LCM) did not predict allocation to asymptotic or non-asymptotic reconstitution groups before (*p* = 0.61, Table 1) or after adjustment for other factors (OR[CDM∶LCM] = 0.93 [0.71, 1.21], *p* = 0.57).

**Table 1:** Characteristics at ART initiation of the two groups of HIV-infected ART-naïve children in the ARROW trial with qualitatively different CD4 reconstitution profiles.

Characteristic	Asymptotic CD4 Reconstitution (Figure 1) (*n* = 914)	Non-Asymptotic CD4 Reconstitution (*n* = 292)	*p*-Value
**Female, ** ***n*** ** (percent)**	474 (52%)	136 (49%)	0.13
**Age (years)**	5.6 (2.3, 9.0)	7.3 (2.8, 10.7)	<0.001
**WHO stage, ** ***n*** ** (percent)**			0.72
1	17 (2%)	3 (1%)	
2	251 (27%)	83 (30%)	
3	514 (56%)	169 (61%)	
4	132 (14%)	37 (13%)	
**CD4 count (cells/µl)** a	350 (170, 646)	401 (155, 837)	0.062
**CD4-for-age (count/healthy count)** a	0.26 (0.14, 0.41)	0.32 (0.14, 0.55)	<0.001
**CD4-for-age ln(count/healthy count)** a	−1.3 (−2.0, −0.9)	−1.1 (−1.9, −0.6)	<0.001
**Weight-for-age ** ***z*** **-score**	−2.3 (−3.3, −1.3)	−2.0 (−3.2, −1.0)	0.023
**Height-for-age ** ***z*** **-score**	−2.5 (−3.5, −1.6)	−2.0 (−3.1, −1.1)	<0.001
**Monitoring randomisation, ** ***n*** ** (percent)**			0.61
LCM	459 (50%)	141 (48%)	
CDM	455 (50%)	151 (52%)	
**First-line ART randomisation, ** ***n*** ** (percent)**			0.024
Arm A	294 (32%)	103 (35%)	
Arm B	325 (36%)	79 (27%)	
Arm C	295 (32%)	110 (38%)	

Data are median (IQR) unless otherwise indicated. *p*-Values are for Fisher's exact (WHO stage and first-line ART randomisation), chi-squared (sex and monitoring randomisation), and Wilcoxon rank sum (continuous variables) tests for a difference between the two groups.

a CD4 count at ART initiation was missing in one child from each group.

Clinically, children with non-asymptotic recovery showed generally worse disease progression than those with asymptotic recovery. 42 (14%) as opposed to 21 (2.3%) switched to second-line therapy (OR 7.13 [4.04, 12.92], *p*<0.001) (none in the first year on ART), and 53 (18%) versus 59 (6.5%) experienced WHO stage 3 or 4 HIV events (OR 3.21 [2.11, 4.87], *p*<0.001).

Post-enrolment viral loads were available in 750 (82%) of the children with asymptotic reconstitution (median [IQR] 4 [3, 7] values available per child) and 200 (68%) of the children with non-asymptotic reconstitution (median [IQR] 3 [2, 6] values available per child). There was some marginal evidence of poorer virological response to ART in the non-asymptotic responders: median (IQR) average log-viral load was 2.07 (1.90, 3.45) compared to 2.03 (1.90, 2.39) in asymptotic responders (*p* = 0.050), and the proportion of viral loads detectable (>80 copies/ml) was 0.33 (0.00, 1.00) compared to 0.25 (0.00, 0.50) (*p* = 0.014).

### Children with Asymptotic CD4 Reconstitution

Figure 2A shows mean CD4-for-age throughout follow-up in this group, who had a median (IQR) age of 5.6 (2.3, 9.0) y and a median (IQR) CD4 count of 345 (176, 655) cells/µl at ART initiation (Table 1). Table 2 gives population average (fixed-effects) parameters as fitted in the nonlinear mixed-effects model, and Figure 2B and 2C illustrate the impact of age on expected CD4-for-age trajectory. There was no evidence of nonlinearity in the relationships between age at ART initiation and CD4 count either at ART initiation or in the long term. From the model, the estimated median (IQR) CD4 count in this group after 3.9 y on ART was 903 (658, 1,281) cells/µl. The proportional recovery rate, *c*, was 0.038 (95% CI: 0.036, 0.040) per week, corresponding to a half-life *t*_1/2_ = ln(2)/*c* (the time taken for half of the ultimate on-ART increase in CD4-for-age from intercept to asymptote to occur) of 18 (95% CI: 17, 19) wk.

**Table 2:** Predictors of CD4-for-age after ART for 914 children with asymptotic CD4 reconstitution.

Variable	Estimate (95%CI)		*p*-Value
	Natural Scale (ln[CD4-for-Age])	Linear Scale (CD4-for-Age)	
**Intercept (CD4-for-age at ART initiation)**	**−1.59 (−1.66, −1.52)**	**0.20 (0.19, 0.22)**	**<0.001**
Age at ART initiation (per year older)	−0.08 (−0.10, −0.06)	0.92 (0.91, 0.94)	<0.001
Weight-for-age at ART initiation (per unit *z*-score higher)	0.16 (0.12, 0.20)	1.17 (1.12, 1.22)	<0.001
**Asymptote (long-term CD4-for-age)**	**−0.21 (−0.26, −0.16)**	**0.81 (0.77, 0.85)**	**<0.001**
Age at ART initiation (per year older)	−0.048 (−0.055, −0.042)	0.95 (0.95, 0.96)	<0.001
Sex (female versus male)	0.11 (0.06, 0.16)	1.11 (1.06, 1.17)	<0.001
ART strategy (Arm B versus Arm A)	0.044 (−0.017, 0.105)	1.04 (0.98, 1.11)	0.16
ART strategy (Arm C versus Arm A)	−0.14 (−0.20, −0.08)	0.87 (0.82, 0.93)	<0.001
*c*	0.038 (0.036, 0.040)	—	<0.001

Results are expressed relative to the reference category (boy, aged 6 y, with weight-for-age *z*-score −2.2 and randomised to first-line ART strategy Arm A [standard of care two-NRTI+NNRTI throughout]). Analysis was carried out on the natural logarithm scale (ln[CD4/expected CD4]), but results are reported both on the natural scale and back-transformed to the linear scale (CD4/expected CD4). Estimated effect sizes of covariates on the baseline (intercept) and the long-term (asymptote) CD4-for-age are additive on the natural log scale, but multiplicative on the linear scale—for example, a 1-y increase in age is associated with a −0.08 unit lower ln(CD4/expected CD4) at ART initiation or, equivalently, a CD4/expected CD4 lower by a factor of exp(−0.08) = 0.92. Similar effect sizes were seen in univariable models. After adjusting for the factors shown, there was no additional predictive value on intercept of sex, WHO disease stage 3 or 4, or first-line ART or monitoring strategy; on asymptote of WHO disease stage 3 or 4, weight-for-age *z*-score, or monitoring strategy, or on *c* of age, sex, WHO disease stage 3 or 4, or ART or monitoring strategy (likelihood ratio tests, *p*>0.01; further details are given in Table S2). Although weight-for-age was not selected in the backwards selection, likelihood ratio tests to assess additional predictors on top of the final multivariable model suggested weight-for-age might be a predictor for *c* (*p* = 0.003 in likelihood ratio test; estimate 9.31×10^−4^ higher per one-unit increase in weight-for-age [95% CI: −7.01×10^−5^, 1.93×10^−3^], i.e., faster proportionate recovery speed in those with higher weight-for-age at ART initiation). However, other effect size estimates were essentially unchanged, and examining closely related covariate models suggests this apparent predictive value may be the result of overfitting. Thus, the final backwards selection is presented as the final model, as described in the Methods.

A strong correlation was estimated between CD4-for-age at ART initiation and in the long term (correlation [95% CI] = 0.37 [0.31, 0.43], *p*<0.001; Figure 2D): that is, children who started ART with higher CD4-for-age achieved higher CD4-for-age in the long term. Interestingly, children with lower CD4-for-age at ART initiation had faster initial recovery (correlation = −0.35 [−0.43, −0.27], *p*<0.001; Figure 2D). These two correlations combined so that those with faster initial recovery also ended up with lower long-term CD4-for-age (correlation = −0.44 [−0.54, −0.33], *p*<0.001; Figure 2D). Such results are consistent with reconstitution processes that are sensitive to a low concentration of CD4 T cells, driving faster recovery in response to lower CD4-for-age, but that are nonetheless unable to reverse significant HIV-related impairment, which is reflected in lower CD4-for-age both before and after treatment. The second important observation was that in multivariable analyses (Table 2), lower CD4-for-age at ART initiation and in the long term were each independently associated with older age at ART initiation (both *p*<0.001; Figure 2B). Lower CD4-for-age at ART initiation was also associated with lower weight-for-age *z*-score (*p*<0.001). Boys achieved lower long-term CD4-for-age values than girls, as did children randomised to zidovudine-containing three-NRTI maintenance (Arm C) compared to those in Arm A (both *p*<0.001). Using backwards selection, we found no covariates whose inclusion in the model for proportional recovery rate *c* substantially improved the fit.

Because CD4 counts in adults starting ART have often been fitted using linear mixed-effects models with a change in slope some weeks after treatment initiation [18], we fitted a similar model in our CD4-for-age data. It proved a substantially worse fit (Akaike information criterion 976 units higher) than the asymptotic model, confirming use of our Equation 1 as more appropriate. Because CD4 recovery in adults is known to be biphasic [32], we also investigated a model that was the sum of two asymptotic functions with different proportional rates of recovery, but were unable to fit it to our data.

**Figure 3:**
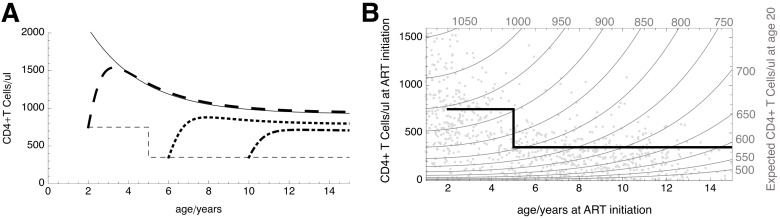
Predicted long-term CD4 counts in children starting ART at different ages and CD4 levels. (A) CD4 trajectories predicted for children starting ART having reached WHO CD4 count thresholds at age 2 (dashed line), 6 (dotted line), or 10 (dot-dashed line) y. The thin dashed line indicates WHO thresholds for ART initiation, and the thin solid line the trajectory in CD4 count with age expected in a healthy child. (B) Expected CD4 count on immunological maturity (estimated at age 20 y) for different ages and CD4 counts at ART initiation. Values at the ends of the grey contour lines indicate expected adult CD4 count in children starting ART at the ages and CD4 counts given on the horizontal and vertical axes. The black line indicates the current WHO CD4 thresholds for ART initiation. Grey point markers show the age and fitted CD4 count at ART initiation of the 914 children on whom the model is based. They indicate at which ages/CD4 counts the model has substantial evidence, and where it represents an extrapolation from the available data.

Using the fitted model parameters, we can predict CD4 trajectories for children with asymptotic reconstitution starting ART at different ages and with different CD4 counts. Figure 3A illustrates predicted trajectories in children starting ART having reached current (2010) WHO ART initiation criteria aged 2, 6, or 10 y. While the 2-y-old is predicted to recover CD4 count well, the 6- and 10-y-olds show progressively impaired recovery. Thus, starting ART at older ages appears to impair long-term CD4 count when treatment is initiated at the WHO CD4 count thresholds for ART initiation, which differ substantially between children aged <5 y and >5 y. From Figure 3B it is apparent that the fixed CD4 threshold for starting ART in children over 5 y old, while satisfactorily reducing risk of disease progression, increasingly limits the potential for optimal CD4 recovery as the child approaches adulthood. In early life, however, relatively large changes in the thresholds for ART initiation have a fairly limited impact on long-term CD4 count.

### Children with Non-Asymptotic CD4 Reconstitution

**Figure 4:**
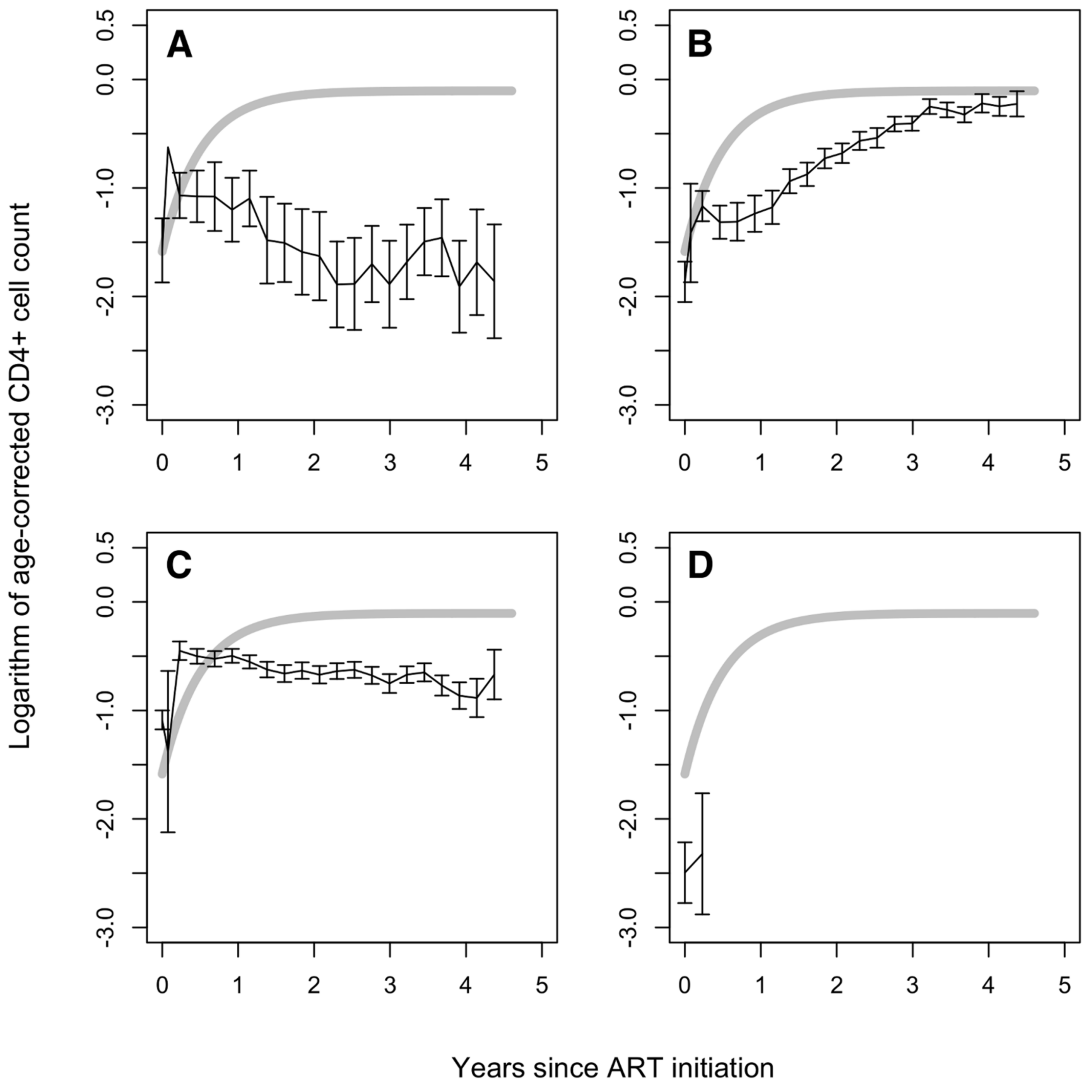
CD4-for-age in children not showing asymptotic reconstitution. Mean CD4 cell count for age with time since ART initiation (error bars: standard error of the mean) for: (A) 23 children with significant (*p*<0.05) decreasing CD4-for-age with time; (B) 79 children with significant (*p*<0.05) increasing CD4-for-age with time but not fitting the asymptotic model; (C) 153 children with no significant change (*p*>0.05) in CD4-for-age with time; and (D) 37 children with only baseline CD4 count available. For comparison to the asymptotic group, grey lines indicate average (fixed-effects, for a 6-y-old girl with weight-for-age *z*-score −2.2) CD4-for-age in the 914 children with asymptotic CD4 reconstitution. In (A–C), data points are shown where >20% of children in each group remained in the trial.

At ART initiation, the 292 children whose CD4 recovery differed qualitatively from the asymptotic reconstitution described above had a median (IQR) age of 7.3 (2.8, 10.7) y and a median CD4 count of 401 (155, 837) cells/µl. Fitting individual linear models to CD4-for-age measurements in each of these children allowed us to identify four subgroups within this population based on a per-child test of significance for the change in CD4-for-age over follow-up (Figure 4; Table 3).

**Table 3:** Characteristics at ART initiation of four subgroups of HIV-infected ART-naïve children who did not show asymptotic CD4-for-age reconstitution on long-term ART (*n  = * 292).

Characteristic	CD4-for-Age Decreasing (*p <* 0.05) (*n = * 23)	CD4-for-Age Increasing (*p <* 0.05) (*n = * 79)	No Significant Change in CD4-for-Age (*p >* 0.05) (*n = * 153)	≤2 Measurements (*n = * 37)	*p*-Value
**Female, *n* (percent)**	12 (52%)	40 (51%)	68 (44%)	16 (43%)	0.59
**Age (years)**	9.2 (6.6, 11.6)	5.5 (2.4, 8.8)	8.1 (3.1, 11.3)	5.4 (1.8, 9.1)	0.009
**WHO stage, *n* (percent)**					0.16
1	0 (0%)	1 (1%)	2 (1%)	0 (0%)	
2	6 (26%)	32 (41%)	39 (25%)	6 (16%)	
3	13 (57%)	39 (49%)	100 (65%)	17 (46%)	
4	4 (17%)	7 (9%)	12 (8%)	14 (38%)	
**CD4 count (cells/µl)^a^**	281 (89, 1013)	360 (83, 737)	479 (230, 980)	130 (22, 671)	0.016
**CD4-for-age (count/healthy count)^a^**	0.28 (0.08, 0.43)	0.28 (0.07, 0.41)	0.40 (0.21, 0.66)	0.12 (0.01, 0.34)	0.002
**CD4-for-age ln(count/healthy count)^a^**	−1.3 (−2.5, −0.9)	−1.3 (−2.7, −0.9)	−0.9 (−1.6, −0.4)	−2.1 (−4.2, −1.1)	0.002
**Weight-for-age *z-*score**	−1.7 (−2.8, −1.4)	−2.0 (−3.2, −0.8)	−1.8 (−2.8, −0.8)	−3.5 (−5.0, −2.4)	0.55
**Height-for-age *z-*score**	−2.1 (−3.4, −1.1)	−2.3 (−3.3, −1.0)	−1.9 (−2.8, −1.2)	−2.7 (−3.4, −1.4)	0.73
**Monitoring randomisation, *n* (percent)**					0.33
LCM	7 (30%)	38 (48%)	71 (46%)	25 (68%)	
CDM	16 (70%)	41 (52%)	82 (54%)	12 (32%)	
**First-line ART randomisation, *n* (percent)**					0.14
Arm A	7 (30%)	35 (44%)	48 (31%)	13 (35%)	
Arm B	10 (43%)	20 (25%)	42 (27%)	7 (19%)	
Arm C	6 (26%)	24 (30%)	63 (41%)	17 (46%)	

Data are median (IQR) unless otherwise indicated. *p*-Values are for Fisher’s exact (categorical variables) or Kruskal-Wallis (continuous variables) tests for a difference between the four groups.

Of these 292 children, 23 (8%) (median [IQR] age: 9.2 [6.6, 11.6] y, CD4 count: 281 [89, 1,013] cells/µl, and CD4-for-age: 0.28 [0.08, 0.43], all at ART initiation) had a significant (*p*<0.05) decline in CD4-for-age over follow-up. These children's mean CD4-for-age values showed an initial, small response to treatment in the first few weeks on ART (Figure 4A). After week 12, however, mean CD4-for-age began to fall, possibly as a result of treatment failure. Finally, as non-response or failure was detected and treatment switched to second-line ART, the CD4-for-age began to recover again. Of note, in univariable tests (Table S1) these children differed from the larger group with asymptotic CD4 reconstitution only in their older age (*p* = 0.002). There was no evidence of between-group differences in other parameters, including CD4-for-age at ART initiation, and monitoring and ART strategy randomisations (*p*>0.08). Clinical progression was worse than in the asymptotic group: five children (22%) switched to second-line treatment (none in the first year on ART), 10 (43%) experienced WHO stage 3 or 4 events, and six (26%) died (all *p*<0.001 for comparison to the large asymptotic group; Table S1). Viral loads were available in 11 children in this group (48%; median [IQR] 2 [1.5, 3] measurements per child). These children had poorer virological control than asymptotic responders: average log-viral load was higher (median [IQR] 4.52 [2.02, 4.76] RNA copies, *p* = 0.014) and a greater proportion of viral loads were detectable (1.00 [0.83, 1.00], *p*<0.001).

79 (27%) of the 292 children (median [IQR] age: 5.5 [2.4, 8.8] y, CD4 count: 360 [83, 737] cells/µl, and CD4-for-age: 0.28 [0.07, 0.41] at ART initiation) showed continuing improvement in CD4-for-age with time, characterised by a significant (*p*<0.05) positive linear slope (Figure 4B). Like the children with asymptotic reconstitution, these children's CD4-for-age increased with time on ART. Unlike the asymptotic group, however, their CD4-for-age increased more slowly and continuously over 4–5 y on ART, although it is possible that CD4 count will plateau in the years following the last available measurement. These children may be showing CD4 recovery that is slower for reasons connected to the mechanism of recovery, and/or to qualitatively different functional impairments at ART initiation. Compared to the large group with asymptotic reconstitution (Table S1), there was evidence in univariate comparisons that these children had lower (less severe) WHO disease stage (*p* = 0.0048 for both stage 3 and stage 4 versus stage 2) and were more likely to be in Arm A and less likely to be in Arm B (*p* = 0.024). Other characteristics were similar (*p*>0.05). Clinical progression was worse than in the asymptotic group: 21 (27%) children switched to second-line therapy (none in the first year on ART), and 16 (20%) experienced WHO stage 3 or 4 HIV events (both *p*<0.001), although only one child died (Table S1). 60 (76%) of these children had at least one viral load recorded, with a median (IQR) 5 (2, 9) measurements per child. Their virological response was similar to that of the asymptotic group: median [IQR] average log-viral load was 1.96 (1.84, 3.10) (*p* = 0.70), and the proportion of viral loads detectable was 0.17 (0.00, 0.53) (*p* = 0.60).

Linear models in 153 (52%) of the 292 children (median [IQR] age: 8.1 [3.1, 11.3] y; CD4 count: 479 [230, 980] cells/µl; CD4-for-age: 0.40 [0.21, 0.66] at ART initiation) showed no evidence of a significant change in CD4-for-age over the first 4–5 y on ART (*p*>0.05) (Figure 4C). These children started ART with a higher CD4 count and CD4-for-age (both *p*<0.001 versus those with asymptotic CD4 reconstitution) and had a small, fast increase in CD4-for-age in the first few weeks following ART, compatible with redistribution of memory CD4 T cells. Following this initial increase, however, CD4-for-age showed no further recovery over 4–5 y of follow-up. Moreover, when ages, weights-for-age, and CD4 counts in these children were used to predict their long-term CD4-for-age according to the nonlinear mixed-effects model for asymptotic reconstitution described above, we found a median (IQR) predicted long-term CD4-for-age of −0.39 (−0.21, +0.00)—somewhat higher than the steady level observed (around −0.5). While these children did show some response to treatment, they seemed to be less able to reconstitute CD4 cells in the long term than the asymptotic group. In addition to their higher CD4-for-age at ART initiation, in univariate comparisons (Table S1) these children were older, heavier, and taller than the large group with asymptotic CD4 reconstitution (*p*<0.001). Clinical progression was worse than in the asymptotic responders: 16 children (10%) switched to second-line therapy (*p*<0.001), 21 (14%) experienced WHO stage 3 or 4 HIV events (*p* = 0.004), and eight died (5.2%, *p*<0.001). (ORs for clinical progression are given in Table S1.) 113 (74%) of these children had viral loads recorded, with a median [IQR] 4 (2, 7) measurements available per child. Their virological response was again similar to that of the asymptotic group: median (IQR) average log-viral load was 2.03 (1.90, 3.18) (*p* = 0.28), and the proportion of viral loads detectable was 0.33 (0.00, 0.75) (*p* = 0.18).

Finally, 37 (13%) of the 292 children (median [IQR] age at ART initiation: 5.4 [1.8, 9.1] y) had very low baseline CD4 counts (median [IQR] 130 [22, 671] cells/µl)—and thus low CD4-for-age (median [IQR] 0.12 [0.02, 0.41])—and ≤1 CD4 measurements available after ART initiation (Figure 4D). 32 of these children died, and five were lost to follow-up, early in the trial.

## Discussion

At present it is recommended that children initiating ART should continue treatment for life. Understanding the long-term impact of treatment initiation at different CD4 counts and ages is therefore essential to ensure that children reach adulthood with immune systems as intact as possible. We have conducted an exploratory study characterising qualitatively and quantitatively different CD4 responses to ART in a large sample of children initiating ART. The analysis, which was not pre-planned or specified in the trial protocol, has allowed us to identify subpopulations of children who respond in qualitatively different ways to ART over the long term.

The majority of children (76%) had asymptotic CD4 reconstitution following ART initiation, with an initial steep increase in CD4-for-age that slowed with a half-life of ∼18 wk, tending towards a constant level over the long term of (for a child of average demographics) ∼80% of the CD4 count expected in an HIV-uninfected child of the same age. CD4-for-age both before ART and in the long term were higher for children starting ART at younger ages, and long-term CD4 reconstitution was better in children with higher CD4-for-age at ART initiation. These results in resource-limited settings reiterate findings in European cohorts [19],[33], and demonstrate the need for earlier diagnosis in all settings, the value of testing children at multiple points of access to healthcare services, and the importance of early availability of ART at minimum at the recommended thresholds for ART initiation. Of note, we found no evidence in these more sophisticated models that CD4 reconstitution (asymptotic or non-asymptotic recovery, or any model parameter) depended on whether children received ART with or without CD4 monitoring. This finding is similar to that of the main randomised comparison [20] and contrasts with findings in adults, in whom there is evidence that CD4 monitoring has benefits for long-term immune reconstitution [9].

Unexpectedly, none of the pre-ART factors considered, nor the randomised groups, were associated with the parameter characterising speed of proportional CD4 recovery. Although this might at first appear inconsistent with previous studies showing faster recovery in younger children [33]–[35], the measure of recovery used elsewhere has generally been an absolute one rather than the proportional rate (*c*), which we used here. Thus, absolute reconstitution may be faster in younger children in that a given increase in CD4 count or percentage takes less time, but a given proportion of the total long-term improvement achieved may still occur in the same time, regardless of age. Nonetheless, we did find significant inter-individual variability in proportional recovery rate that was unexplained by the pre-ART factors studied. Genetic differences between children and/or viral strains may be one explanation for this, and the qualitative composition of the CD4 count when ART began—for example, the percentages of activated and proliferating cells—may also play a role.

As in European/US children, we found that the asymptotic model was a better fit to the data from African children than the two-slope linear mixed-effects model that is optimal in adults [18]. This finding adds further weight to the hypothesis that CD4 recovery in children differs qualitatively and mechanistically from that in adults, perhaps reflecting greater involvement of the naïve T cell pool and greater dependence on thymopoiesis; this finding is supported by the difference in the impact of CD4 monitoring on long-term reconstitution in adults [9] compared to children [20]. Some of the conclusions from adult studies do, however, apply also in children. For example, like the children studied here, adults with low CD4 counts at ART initiation have been observed to have generally lower CD4 counts 3 y later [36].

Using parameters obtained from the nonlinear mixed-effects model for asymptotic CD4 reconstitution, we were able to predict CD4 trajectories for children starting ART at different ages and with different CD4 counts, reflecting the response that would be expected from the majority of children initiating ART. Qualitatively, our findings are similar to those obtained in previous work on European and Brazilian children [19], but the far larger numbers in the present study now provide much greater certainty and also demonstrate that factors such as co-infection burden, malnutrition, and trial recruitment criteria do not appear to cause large differences in the CD4 response to ART. From Figure 3B, predicted CD4 count at age 20 y (i.e., in adulthood) can be estimated for every combination of age and CD4 count at ART initiation. Although our predictions extrapolate the data forward in time, the wide age and CD4 range at ART initiation, and the stability of results compared to analysis through 3 y, suggest that the extrapolations are reasonable. Nevertheless, further analysis of long-term CD4 response in adolescents will be important as validation. The figure shows how starting ART at the same CD4 count but at progressively older age is increasingly detrimental to long-term immunological health. For example, to achieve a CD4 count of >700 cells/µl at 20 y of age, children under 8 y need to start ART with a CD4 count >223 cells/µl, but a 10-y-old would need to start before CD4 count falls below 347 cells/µl, and a 12-y-old before reaching 557 cells/µl. In contrast, a 2-y-old would need only a CD4 count >96 cells/µl, and a 5-y-old, CD4 count >130 cells/µl. To optimise immune reconstitution when children reach adulthood, our findings suggest that ART may be warranted in vertically HIV-infected children who are still ART-naïve at ages over 10 y, regardless of CD4 count. In young children, however, current guidelines designed to minimise risk of disease progression also preserve the potential for good CD4 counts in the long term.

Clearly, the aim of an ART strategy for an HIV-infected child is not limited to maximising CD4 count, but is a matter of balancing overall immunological health—encompassing lymphocyte dynamics, population structure and receptor diversity, and the effectiveness of the immune response overall—against short-term side effects, long-term toxicity, regimen durability (particularly in resource-limited settings where frequently only two regimens are available), cost, treatment adherence, and social factors. Even a CD4 count as low as 500 cells/µl is above the 2.5th centile for CD4 count in a population of healthy adults, raising questions about the appropriateness of aiming for a long-term CD4 count as high as, for example, 700 cells/µl, especially where multiple other factors might argue against ART. However, even relatively small reductions from normal CD4 counts in HIV-infected patients have been associated with increased risk of cardiovascular disease and malignancies [37]–[39]. We also note that our predictions are of CD4 response expected on average in HIV-infected children starting ART. While an increase in the CD4 count threshold for ART initiation might result in an only marginally higher long-term count for the “average” child, the shift in the whole distribution of CD4 counts to higher levels results in fewer children being likely to experience lower counts.

Interestingly, we found slightly, but significantly, lower long-term CD4-for-age values in children receiving three-NRTI maintenance (including zidovudine) (Arm C), despite no differences in CD4 cell percentage over the long term [20] and no difference in the proportion achieving asymptotic response compared to standard two-NRTI+NNRTI treatment. One explanation is the known haematological toxicity of zidovudine, which may modestly suppress all components of the white blood count, leaving proportionate (percentage) recovery unchanged. Whether these small differences in long-term asymptote (∼13% reduction compared to Arm A) are clinically important is unknown. However, we also found that children randomised to Arm B (four-drug, two-class induction followed by standard two-NRTI + NNRTI maintenance) were more likely to have asymptotic than non-asymptotic reconstitution profiles, a novel finding not identified by the standard comparison of mean responses [20]. This supports the potential of enhanced initial treatment to improve long-term CD4 responses.

Approximately one-quarter of children did not have asymptotic CD4 reconstitution. The fact that subgroups within the population had very different patterns of reconstitution, and varied in both CD4 count and age at ART initiation, highlights the issue of modelling one single population mean response across qualitatively different types of reconstitution. Those who had a constant or increasing CD4-for-age following ART had a higher CD4-for-age at ART initiation. We suggest that the differences between these children and those showing asymptotic reconstitution may arise from different reconstitution mechanisms, or qualitatively different CD4 T cell populations at ART initiation. Other children whose CD4 count declined on ART had similar CD4-for-age at ART initiation but were older than the group with asymptotic CD4 reconstitution. Children with ≤2 CD4 measurements after ART initiation had had very low CD4-for-age values when ART was begun; most died early in the trial. Factors affecting reconstitution profiles in these 292 children may have included clinical factors, adherence, and viral dynamics, and investigating these and other factors/mechanisms influencing CD4 recovery in children with atypical CD4 responses will provide interesting directions for further research. Our findings regarding the different CD4 reconstitution groups should also be validated in independent studies.

Children in each of the non-asymptotic recovery groups experienced more regimen switching, WHO disease stage 3 and 4 events, and deaths than the large group of asymptotic responders. While this is an interesting and important observation, it does not allow any conclusions about causation to be drawn. On the one hand, it is possible that more frequent clinical events might have impaired children's CD4 recovery and led to their different reconstitution profiles. Equally, however, since children in the non-asymptotic groups had CD4-for-age values below those of the asymptotic responders at almost all time points (Figure 4), it is unsurprising that they experienced more regimen switching, clinical events, and deaths. What can be stated is that, since logistic regression models showed little absolute difference in age-corrected CD4 counts between the two groups at ART initiation, the CD4 count when ART begins does not explain the substantial difference between asymptotic and non-asymptotic recovery.

One of the limitations of this study was the lack of systematic viral load measurements, which have only been retrospectively assayed in subsets of children at restricted time points [20] (further work ongoing). Previous work suggests that viral loads before ART are likely to predict CD4 count at ART initiation (though not in the long term) [19], and that joint models incorporating CD4 count and viral load are superior to univariate models of CD4 response only [40]. In the absence of systematic viral load measurements on all children, we were not able to assess conclusively whether patterns of viraemia could be contributing to the patterns of CD4 response. Nevertheless, the incomplete results that were available suggested that the children with falling CD4-for-age (Figure 4A, 23 children) had poorer virological control than the group with asymptotic recovery. However, children with slowly increasing (Figure 4B, 79 children) or constant (Figure 4C, 153 children) CD4-for-age had similar virological responses to the asymptotic responders. There were examples of viral rebound in all response groups. Hence, although virological control may be an important factor for children with falling CD4 counts, it does not appear to explain the differences in CD4 responses between the asymptotic group and the two major non-asymptotic groups.

A limitation of the modelling approach is that uncertainty as to children's group allocation to asymptotic or non-asymptotic recovery is not accounted for. This problem is illustrated by the observation that an earlier analysis—of data up to 1 April 2011 (i.e., missing the last year of the trial)—reversed the allocations of a small number of children. The uncertainty in allocation could be modelled using mixture models or latent class models, and this will be an important further step in developing this kind of analysis. However, the robustness of allocation in the vast majority of children when data from the last year of the trial were included or excluded suggests it is unlikely that failing to model the uncertainty has materially affected our conclusions. In previous work, we fitted a nonlinear mixed-effects model with an asymptotic recovery function to a whole cohort of HIV-infected children starting ART. In spite of modelling recovery as asymptotic in all children, the same conclusion was reached: that age affects CD4 count both before ART and in the long term, and that there is an additional correlation between unexplained differences in counts at ART initiation and in the long term [19]. Thus, although parameter estimates might be revised slightly by a more rigorous model, it seems likely that our conclusions would remain unchanged. The allocation of children to asymptotic versus non-asymptotic recovery groups also raises a problem with predictions: while predictions like those illustrated in Figure 3B are helpful for children who will go on to experience asymptotic recovery, they are less relevant to those with different recovery profiles. This is a particular example of a general and widespread problem with using population-level responses to inform treatment guidelines. In this context, at the level of individual children, it highlights the challenge of identifying baseline predictors of non-asymptotic recovery so that those children can be treated differently.

In conclusion, our analysis of data from a large cohort of HIV-infected, ART-naïve African children initiating ART provides important information to inform clinical practice by considering long-term CD4 reconstitution in response to therapy in resource-limited settings, and the roles of age and CD4 count at ART initiation in determining this response. Given increased availability of simpler ART formulations and regimens for children, we have reinforced the importance of considering long-term immunological health, as well as short-term disease progression, in the decision to initiate ART and the development of treatment guidelines. Our data suggest that even though the short-term risks of morbidity and mortality are greater in younger untreated children, survivors have excellent potential for immune reconstitution when they initiate ART. In contrast, whilst not subject to the same immediate risks of disease progression/death, older children who wait to initiate ART may never reconstitute immunologically to the same degree. The current CD4 count thresholds for ART initiation are predominantly based on these short-term risks, and in older children could lead to delays in ART administration that might compromise immune reconstitution in adulthood.

Our results indicate that although younger ART-naïve children are at high risk of disease progression, provided they start ART following current WHO/Paediatric European Network for Treatment of AIDS/US Centers for Disease Control and Prevention guidelines, they have good potential for achieving high CD4 levels in later life. However, to attain maximum immune reconstitution in older children, particularly those >10 y, ART may need to be initiated regardless of CD4 cell count. The approach taken in this study to characterise long-term immunological outcomes has the potential, by identifying qualitatively different CD4 recovery profiles, to improve our understanding of the factors that determine children's CD4 counts on ART and provide input into future management guidelines in both resource-rich and resource-limited settings.

## Supporting Information

Figure S1 **Relationship between very low CD4 count and CD4-for-age** ***z*** **-score in children of different ages.**
(PDF)

Table S1 **Univariate comparisons between individual non-asymptotic response subgroups and the large (** ***n*** **= 914) group of asymptotic responders.**
(DOC)

Table S2 **Additional predictors of CD4-for-age profiles in the asymptotic recovery group.**
(DOC)

## Abbreviations

ARROW: Antiretroviral Research for Watoto
ART: antiretroviral therapy
CDM: clinically driven monitoring
IQR: interquartile range
LCM: laboratory and clinical monitoring
NNRTI: non-nucleoside reverse transcriptase inhibitor
NRTI: nucleoside reverse transcriptase inhibitor
OR: odds ratio
