# Supplementary figures and images for: Predicting Patterns of Long-Term CD4 Reconstitution in HIV-Infected Children Starting Antiretroviral Therapy in Sub-Saharan Africa: A Cohort-Based Modelling Study
Source: PLoS Med. 2013 Oct 29;10(10):e1001542. doi: 10.1371/journal.pmed.1001542 (PMC3812080; doi:10.1371/journal.pmed.1001542)

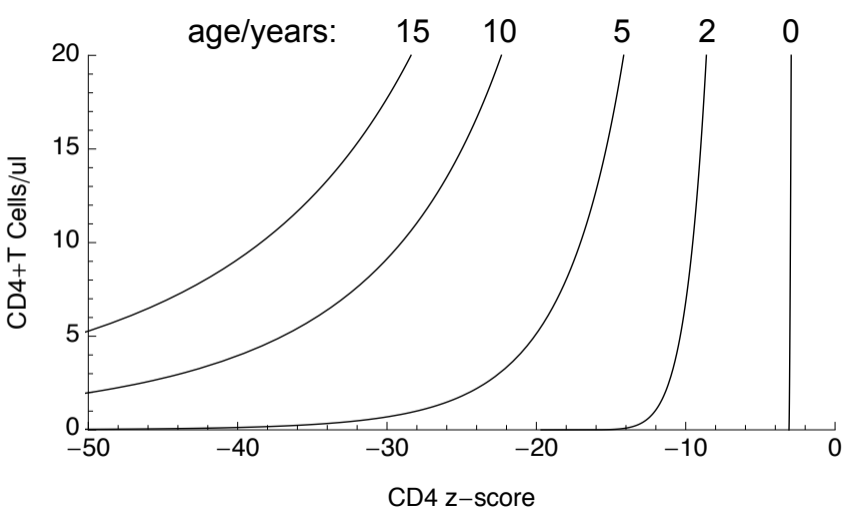

Supplement: Figure S1 — Relationship between very low CD4 count and CD4-for-age z -score in children of different ages. (PDF) [file pmed.1001542.s001.pdf]
