# Supplementary material for: Predicting Patterns of Long-Term CD4 Reconstitution in HIV-Infected Children Starting Antiretroviral Therapy in Sub-Saharan Africa: A Cohort-Based Modelling Study
Source: PLoS Med. 2013 Oct 29;10(10):e1001542. doi: 10.1371/journal.pmed.1001542 (PMC3812080; doi:10.1371/journal.pmed.1001542)
Supplement: Table S1 — Univariate comparisons between individual non-asymptotic response subgroups and the large ( n = 914) group of asymptotic responders. (DOC) [file pmed.1001542.s002.doc]

**Table S1: Univariate comparisons between individual non-asymptotic response sub-groups and the large (n=914) group of asymptotic responders.**

| **Characteristics** |  | **CD4-for-age decreasing (n=23) (value (95% CI); p-value)** | **CD4-for-age increasing (n=79) (value (95% CI); p-value)** | **No significant change in CD4-for-age (n=153) (value (95% CI); p-value)** | **≤2 measurements (n=37) (value (95% CI); p-value)** |
| --- | --- | --- | --- | --- | --- |
| Pre-ART age (years) |  | +3.2 (+1.1, +5.1); 0.002 | +0.13 (-0.67,+0.93); 0.74 | +1.6 (+0.8, +2.4); <0.001 | -0.12 (-1.32, +1.27); 0.84 |
| Pre-ART CD4 count (cells/μL) |  | -25 (-167, +115); 0.70 | -7 (-84,+71); 0.80 | +134 (+69, +199); <0.001 | -111 (-220, -11); 0.02258 |
| Pre-ART CD4-for-age (count/healthy count) |  | +0.01 (-0.09, +0.10); 0.83 | +0.00 (-0.05, +0.05); 1.00 | +0.14 (+0.09, +0.18); <0.001 | +0.10 (-0.17, -0.03); 0.002 |
| Pre-ART CD4-for-age ln(count/healthy count) |  | +0.05 (-0.42, +0.51); 0.83 | +0.00 (-0.22, +0.22); 1.00 | +0.46 (+0.31, +0.60); <0.001 | -0.64 (-1.24, -0.22); 0.002 |
| Pre-ART weight-for-age z-score |  | +0.29 (-0.28, +0.88); 0.30 | +0.29 (-0.08, +0.66); 0.14 | +0.51 (+0.25, +0.77); <0.001 | -1.2 (-1.8, +0.7); <0.001 |
| Pre-ART height-for-age z-score |  | +0.40 (-0.31, +1.01); 0.25 | +0.34 (-0.01, +0.69); 0.060 | +0.52 (+0.27, +0.76); <0.001 | -0.10 (-0.58, +0.38); 0.69 |
| Sex | female vs male | 0.99 (0.39, 2.47); 1.00 | 1.05 (0.64, 1.71); 0.91 | 1.35 (0.94, 1.93); 0.10 | 0.74 (0.36, 1.53); 0.40 |
| WHO stage | 3 vs 2 | 0.95 (0.29, 2.70); 1.00 | 1.68 (0.99, 2.82); 0.048 | 0.80 (0.52, 1.21); 0.28 | 1.66 (0.58, 5.82); 0.37 |
|  | 4 vs 2 | 0.79 (0.18, 3.87); 0.74 | 2.40 (1.00, 6.62); 0.048 | 1.71 (0.84, 3.71); 0.15 | 5.30 (1.76, 19.22); <0.001 |
| Monitoring randomisation | CDM vs LCM | 0.43 (0.15, 1.13); 0.089 | 0.92 (0.56, 1.49); 0.73 | 0.86 (0.60, 1.23); 0.43 | 0.50 (0.23, 1.06); 0.062 |
| First-line ART randomisation: | B vs A | 0.77 (0.25, 2.29); 0.63 | 1.93 (1.06, 3.62); 0.024 | 1.26 (0.79, 2.02); 0.31 | 0.49 (0.16, 1.34); 0.17 |
|  | C vs A | 1.17 (0.33, 4.27); 1.00 | 1.46 (0.82, 2.64); 0.18 | 0.76 (0.49, 1.17); 0.21 | 1.23 (0.54, 2.82); 0.70 |
| Switched to second-line therapy |  | 11.7 (2.1, 37.1); <0.001 | 15.3 (7.5, 31.4); <0.001 | 4.95 (2.35, 10.24); <0.001 | - |
| WHO class 3/4 events |  | 11.1 (4.2, 28.7); <0.001 | 3.67 (1.86, 6.93); <0.001 | 2.30 (1.28, 4.00); 0.004 | 2.80 (0.91, 7.18); 0.035 |
| Deaths |  | 44.7 (11.2, 174.4); <0.001 | 1.66 (0.04, 13.19); 0.49 | 7.13 (2.22, 23.47); <0.001 | 222 (778, 4110); <0.001 |

The upper rows of the table (pre-ART age to pre-ART height-for-age) refer to comparisons of continuous variables. The values given are estimated (95% confidence interval, 95% CI) differences between each non-asymptotic response group and the large, asymptotic group, followed by a p-value for a Wilcoxon rank sum test with the null hypothesis of zero difference. For example, the age of children with decreasing CD4-for-age was estimated to be 3.2 (95% CI 1.1 to 5.1) years older than the children with asymptotic reconstitution, corresponding to a p-value of 0.00182. The lower rows (sex to first-line ART randomisation) refer to categorical variables, and the values given are an odds ratio with 95% CI and p-value for Fisher’s exact test, with the null hypothesis of odds ratio 1. None of the children with only one or two CD4 counts available
