# Supplementary material for: Predicting Patterns of Long-Term CD4 Reconstitution in HIV-Infected Children Starting Antiretroviral Therapy in Sub-Saharan Africa: A Cohort-Based Modelling Study
Source: PLoS Med. 2013 Oct 29;10(10):e1001542. doi: 10.1371/journal.pmed.1001542 (PMC3812080; doi:10.1371/journal.pmed.1001542)
Supplement: Table S2 — Additional predictors of CD4-for-age profiles in the asymptotic recovery group. (DOC) [file pmed.1001542.s003.doc]

**Table S2: Additional predictors of CD4-for-age profiles in the asymptotic recovery group.**

|  |  | **Estimate** | **Standard Error** | **p-value (Likelihood ratio test)** |
| --- | --- | --- | --- | --- |
| **Predictors of intercept** |  |  |  |  |
| Sex | Female | 0.137 | 0.068 | 0.018 |
| WHO stage | Stage 3 | 0.0679 | 0.0659 | 0.079 |
|  | Stage 4 | -0.121 | 0.099 | 0.046 |
| Monitoring strategy | CDM | 0.104 | 0.065 | 0.029 |
| First-line ART strategy | Arm-B | -0.0409 | 0.0823 | 0.21 |
|  | Arm-C | -0.0791 | 0.0845 |  |
| **Predictors of asymptote** |  |  |  |  |
| WHO stage | Stage 3 | 0.0142 | 0.0257 | 0.12 |
|  | Stage 4 | 0.0809 | 0.0367 | 0.012 |
| Pre-ART weight-for-age | Per unit higher | -0.0220 | 0.0083 | 0.034 |
| Monitoring strategy | CDM | -0.0197 | 0.0255 | 0.078 |
| **Predictors of c** |  |  |  |  |
| Pre-ART age | Per year older | 7.64×10^-4^ | 2.50×10^-4^ | 0.034 |
| Sex | Female | 8.46×10^-4^ | 1.81×10^-3^ | 0.18 |
| WHO stage | Stage 3 | 1.53×10^-3^ | 1.71×10^-3^ | 0.11 |
|  | Stage 4 | -9.68×10^-4^ | 2.24×10^-3^ | 0.089 |
| Pre-ART weight-for-age | Per unit higher | 9.31×10^-4^ | 5.11×10^-4^ | 0.003 |
| Monitoring strategy | CDM | -2.29×10^-3^ | 1.70×10^-3^ | 0.024 |
| First-line ART strategy | Arm-B | -5.65×10^-3^ | 2.18×10^-3^ | 0.020 |
|  | Arm-C | -2.94×10^-3^ | 2.33×10^-3^ |  |

We examined the effects of including additional predictors of parameter values in the nonlinear mixed-effects model described in Table 2. Predictive effects on each of the three model parameters were included one at a time. The table gives the estimated and standard error effect sizes, and the results of likelihood ratio tests comparing each extended model to the model in Table 2.
